# Nucleolar protein 10 (NOP10) predicts poor prognosis in invasive breast cancer

**DOI:** 10.1007/s10549-020-05999-3

**Published:** 2020-11-08

**Authors:** Khloud A. Elsharawy, Maryam Althobiti, Omar J. Mohammed, Abrar I. Aljohani, Michael S. Toss, Andrew R. Green, Emad A. Rakha

**Affiliations:** 1 Nottingham Breast Cancer Research Centre, Division of Cancer and Stem Cells, School of Medicine, University of Nottingham Biodiscovery Institute, University Park, Nottingham, UK; 2 Faculty of Science, Damietta University, Damietta, Egypt; 3 Department of Clinical Laboratory Science, College of Applied Medical Science, Shaqra University, Shaqra, Saudi Arabia; 4 Division of Cancer and Stem Cell, University of Nottingham, City Hospital Campus, Hucknall Road, Nottingham, NG5 1PB UK

**Keywords:** NOP10, Invasive breast cancer, Nucleoli, Prognosis

## Abstract

**Purpose:**

Nucleolar protein 10 (NOP10) is required for ribosome biogenesis and telomere maintenance and plays a key role in carcinogenesis. This study aims to evaluate the clinical and prognostic significance of NOP10 in breast cancer (BC).

**Methods:**

*NOP10* expression was assessed at mRNA level employing the Molecular Taxonomy of Breast Cancer International Consortium (METABRIC) (*n* = 1980) and Cancer Genome Atlas (TCGA) BC cohorts (*n* = 854). Protein expression was evaluated on tissue microarray of a large BC cohort (*n* = 1081) using immunohistochemistry. The correlation between NOP10 expression, clinicopathological parameters and patient outcome was assessed.

**Results:**

NOP10 expression was detected in the nucleus and nucleolus of the tumour cells. At the transcriptomic and proteomic levels, NOP10 was significantly associated with aggressive BC features including high tumour grade, high nucleolar score and poor Nottingham Prognostic Index. High NOP10 protein expression was an independent predictor of poor outcome in the whole cohort and in triple-negative BC (TNBC) class (*p* = 0.002 & *p* = 0.014, respectively). In chemotherapy- treated patients, high NOP10 protein expression was significantly associated with shorter survival (*p* = 0.03) and was predictive of higher risk of death (*p* = 0.028) and development of distant metastasis (*p* = 0.02) independent of tumour size, nodal stage and tumour grade.

**Conclusion:**

High NOP10 expression is a poor prognostic biomarker in BC and its expression can help in predicting chemotherapy resistance. Functional assessments are necessary to decipher the underlying mechanisms and to reveal its potential therapeutic values in various BC subtypes especially in the aggressive TNBC class.

**Electronic supplementary material:**

The online version of this article (doi:10.1007/s10549-020-05999-3) contains supplementary material, which is available to authorized users.

## Introduction

Invasive breast cancer (BC) is a heterogeneous disease with a spectrum of different molecular and morphological subtypes that are variable in behaviour and response to therapy [1]. Morphological features such as histological grade have a prognostic value and their assessment help in treatment decisions of BC patients [2]. Nucleolar aberration either in size, shape, or number [3], has been associated with poor outcomes in BC [4]. Alterations in the nucleolus during tumourigenesis usually take place as a consequence of elevating ribosomal biogenesis to subtend the high demand for proteins in cancer cells which is highly required for their proliferation [5, 6]. RNA modifications occur in the nucleolus and require hundreds of small nucleolar RNAs (snoRNAs), as well as multi-component protein complexes which are collectively known as small nucleolar ribonucleoprotein (snoRNP) complexes [7]. Dysregulation of snoRNPs can influence the development and progression of various human diseases such as Prader Willi syndrome, some metabolic stress disorders, and several types of cancer [8–11].

These complexes include two classes, C/D box snoRNPs and H/ACA box snoRNPs, which enhance fundamental processes in ribosomal RNA modification [12–14]. H/ACA RNPs are composed of four main conserved proteins, including Nucleolar Protein Family A, Member 3 (NOP10), DKC1 (Dyskerin), Nucleolar Protein Family A, Member 2 (NHP2) and Nucleolar Protein Family A, Member 1 (GAR1) [12]. DKC1 expression is upregulated in a number of human cancers including BC and its high levels are associated with poor prognosis through the disruption of several cellular processes including telomere maintenance, mitosis, transcription, and RNA processing [15–17]. However, it is unclear whether the underlying mechanisms are related only to DKC1 dysregulation or perhaps result from a disrupted function of other proteins in the H/ACA RNPs box. NOP10, also known as NOLA3, plays critical roles in diverse processes, including the processing of ribosomal RNAs, modification of spliceosome small nuclear RNAs, and stabilization of telomerase [18]. It forms a core trimer with DKC1 and NHP2 where they closely interact with each other and are involved in the metabolic stability of RNAs [19, 20].

The current study aims to examine the molecular and prognostic value of NOP10 at genomic, transcriptomic, and proteomic levels in BC by assessing its association with clinicopathological parameters and patient outcome using several large cohorts and datasets with the emphasis on the different BC molecular subtypes.

## Materials and methods

### Study cohorts

When the members in H/ACA RNPs box were analysed at the transcriptomic levels in both the Molecular Taxonomy of Breast Cancer International Consortium (METABRIC) cohort (*n* = 1980) and The Cancer Genome Atlas (TCGA) BC dataset (*n* = 854), NOP10 was highly associated with aggressive tumour features and worse patient outcome, compared to others. Therefore, NOP10 was selected for this study. The METABRIC cohort (*n* = 1980) was used to evaluate NOP10 gene copy number (CN) aberrations and mRNA expression [21]. The TCGA BC dataset and the Breast Cancer Gene Expression Miner online dataset v4.3 (https://bcgenex.ico.unicancer.fr) were used as external validation resources of NOP10 mRNA expression [22].

NOP10 protein expression was evaluated on well-characterized large series (*n* = 1081) of BC patients (Nottingham Series) as previously described [23]. Briefly, patients were presented at Nottingham City Hospital between 1999 and 2006. Clinical information and tumour characteristics including patient’s age at diagnosis, histological tumour type, grade, tumour size, lymph node status, Nottingham Prognostic Index (NPI), and lymphovascular invasion (LVI), were available. Moreover, the data of the nucleolar scoring of Nottingham and TCGA breast cancer datasets were available as previously published [4]. Outcome data were obtained and these included BC specific survival (BCSS), defined as time (in months) from the date of primary surgical treatment to the time of death by BC, and distant metastasis-free survival (DMFS) defined as the time (in months) from the surgery until the first event of distant metastasis. Post-operative treatment was offered according to the institutional protocols. Data related to the expression of basic BC markers were also available, including oestrogen receptor (ER), progesterone receptor (PR), and human epidermal growth factor 2 (HER2) [24–26]. In addition, DKC1 protein expression level was assessed on the same cohort. Endocrine therapy was given to patients who had ER-positive (ER +) tumours with high NPI scores (> 3.4), while no adjuvant therapy was given to patients with ‘good’ NPI scores (≤ 3.4). Post-menopausal patients with ‘moderate’ or ‘poor’ NPI scores were given hormonal therapy, while premenopausal patients with moderate and poor NPI scores were subject to chemotherapy. Classical treatment of cyclophosphamide, methotrexate, and fluorouracil (CMF) was used as a therapy for patients presented with the absence of ER expression and clinically fit to receive chemotherapy. None of the patients in the current study cohort received neoadjuvant therapy. The clinicopathological parameters for the METABRIC and Nottingham series are summarized in (Supplementary Table S1).

### NOP10 protein expression evaluation

**Fig. 1:**
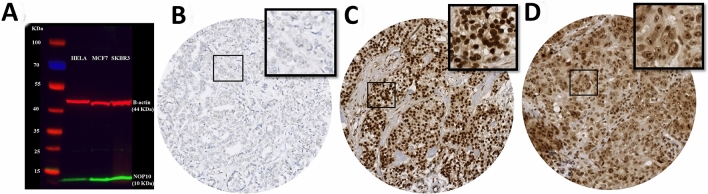
**a** Western blotting of anti-NOP10 antibody in HELA, MCF7 and SKBR3 cell lysates showing a single specific band (lower green band) at expected molecular weight (10KDa). The upper red band represented β-actin (positive control) at 42 kDa, **b** Negative NOP10 IHC expression, **c** Positive NOP10 IHC nuclear expression and **d** Positive NOP10 IHC nucleolar expression in invasive breast cancer TMA cores

Prior to immunohistochemistry (IHC) staining of the tissue sections, the specificity of the anti-NOP10 antibody (EPR8857, Abcam, UK) was validated by Western blotting using cell lysates of HELA, MCF7 and SKBR3 human cell lines obtained from American Type Culture Collection, Rockville, MD, USA. The NOP10 primary antibody was used at 1:1000 dilution. Proteins were detected using IRDye 800CW fluorescent secondary antibodies (1:15 000 dilution, LI-COR Biosciences) and the Odyssey Fc with Image Studio 4.0 (LI-COR Biosciences) was used to visualize the bands. Anti-β-actin primary antibody (Sigma-Aldrich) was used as a loading control (1: 5000). A single specific band for NOP10 protein was observed at the predicted molecular weight (10 kDa) confirming the specificity of the antibody (Fig. 1a).

Tumour samples were previously arrayed (TMAs) using the Grand Master® (3D HISTECH®, Budapest, Hungary) as described previously [27]. IHC staining was performed on 4 μm thick sections using the Novocastra Novolink™ Polymer Detection Systems kit (Code: RE7280-K, Leica, Biosystems, Newcastle, UK). Antigen retrieval was performed in citrate buffer pH 6.0 using a microwave (Whirlpool JT359 Jet Chef 1000 W) for 20 min. Rabbit monoclonal NOP10 was diluted at 1:250 in Leica antibody diluent (RE AR9352, Leica, Biosystems, UK) and incubated for 60 min at room temperature. A negative control was obtained by omitting the primary antibody, while normal renal tissue was used as a positive control according to the manufacturer’s datasheet.

### Scoring NOP10 protein expression

Scoring of NOP10 expression was performed using high-resolution digital images of the stained TMAs, which were obtained via a NanoZoomer scanner (NanoZoomer; Hamamatsu Photonics, Welwyn Garden City, UK) at 20 × magnification and were viewed using Xplore software (Philips, UK). Immunoreactivity staining of the nucleus and prominent nucleolus [4] were evaluated based on a semi-quantitative scoring using a modified histochemical score (H-score), which estimated both the intensity and the percentage of stained tumour cells. A score index of 0, 1, 2, and 3 corresponding to a negative, weak, moderate, and strong staining, respectively, was used for intensity scoring. The percentage (0–100) of positive cells for each intensity was evaluated. The final H-score was calculated by multiplying the percentage of positively stained cells in the tissue by the level of intensity, generating a score ranging between 0 and 300 [28]. All non-representative cores including folded tissue during processing and staining, cores with < 15% of invasive tumour tissue were excluded. Scoring was performed by one observer (K. Elsharawy) blinded of clinicopathological and patients’ outcome data. Moreover, the inter-observer’s reproducibility of the scoring was considered. A subset of cores (10%) was randomly selected and double scored blindly by another observer (M. Althobiti).

This work obtained ethics approval to use the human tissue samples by the North West – Greater Manchester Central Research Ethics Committee under the title; Nottingham Health Science Biobank (NHSB), reference number 15/NW/0685. Informed consent was obtained from all individuals prior to surgery to use their tissue materials in research.

### Statistical analysis

NOP10 expression for proteomic and transcriptomic was categorized using X-tile bioinformatics software version 3.6.1 (School of Medicine, Yale University, New Haven, USA) based on a prediction of BCSS [29]. IBM-SPSS statistical software 24.0 (SPSS, Chicago, IL, USA) was used for statistical analysis. Inter-observer agreement in NOP10 IHC scoring was assessed using intra-class correlation coefficient (ICC). The Chi-square test was performed for correlations between categorical variables. Spearman’s rank correlation coefficient was carried out to examine the association between NOP10 and other related markers. Univariate survival analysis was carried out using Kaplan–Meier curves and log rank test. Cox’s regression models were used for the multivariate survival analysis to adjust for confounding factors. For all tests, *p*-value < 0.05 was considered statistically significant.

This study followed the reporting recommendations for tumour markers prognostic studies (REMARK) criteria [30] (Supplementary Table S2).

## Results

### Genomic and transcriptomic expression of *NOP10*

High *NOP10* mRNA expression (log2 intensity > 11.2) was observed in 493/1968 (25%) of the METABRIC cases. A significant association was observed between high *NOP10* mRNA expression and *NOP10* gene copy number (CN) gain (*p* < 0.0001). High *NOP10* mRNA expression was significantly associated with features characteristic of poor prognosis including younger age, higher tumour grade, poorer NPI scores, hormone receptor negativity, HER2 positivity, TNBC phenotype (all *p* < 0.0001) and advance nodal stage (*p* = 0.001). According to the intrinsic PAM50 subtypes [31], higher expression of *NOP10* mRNA was found mainly in basal-like BC (*p* < 0.0001) (Table 1).

**Table 1:** Association of *NOP10* mRNA expression with clinicopathological parameters in the Molecular Taxonomy of Breast Cancer International Consortium (METABRIC) and in the Cancer Genome Atlas (TCGA) breast cancer series

Parameters	METABRIC			TCGA		
	*NOP10* mRNA		Chi- square (χ2) (p-value)	*NOP10* mRNA		Chi- square (χ2) (*p*-value)
	Low N (%)	High N (%)		Low N (%)	High N (%)	
**Patient age**						
≤ 50	245 (65)	135 (35)	28.5 ** < 0.0001**	180 (83)	36 (17)	6.5 **0.011**
> 50	1202 (78)	345 (22)		525 (90)	59 (10)	
**Tumour size**						
≤ 2 cm	442 (71)	178 (29)	6.4 **0.012**	206 (93)	16 (7)	6.4 **0.011**
> 2 cm	1012 (77)	309 (23)		499 (86)	79 (14)	
**Axillary node stage**						
Negative	1247 (76)	401 (24)	14.9 **0.001**	355 (89)	44 (11)	0.5 0.5
Positive	224 (72)	89 (28)		347 (87)	50 (13)	
**Lympho-vascular Invasion**						
Negative	***N/A***	***N/A***	***N/A***	474 (90)	52 (10)	5.8 (**0.016**)
Positive				231 (84)	43 (16)	
**PAM50 subtypes**						
Luminal A	620 (86)	97 (14)	102.1 ** < 0.0001**	***N/A***	***N/A***	***N/A***
Luminal B	335 (69)	151 (31)				
Basal like	206 (63)	120 (37)				
HER2	151 (64)	85 (36)				
Normal	158 (80)	39 (20)				
**Nottingham prognostic index (NPI)**						
Good	548 (81)	129 (19)	25.5 **<0.0001**	***N/A***	***N/A***	***N/A***
Moderate	799 (73)	295 (27)				
Poor	128 (65)	69 (35)				
**Tumour grade**						
Grade 1	137 (81)	32 (19)	35.4 ** < 0.0001**	80 (96)	3 (4)	25.7 ** < 0.0001**
Grade 2	619 (81)	149 (19)		324 (93)	26 (7)	
Grade 3	649 (69)	295 (31)		270 (82)	61 (18)	
**Nucleolar score**						
Score 1	***N/A***	***N/A***	***N/A***	269 (94)	17(6)	48.2 ** < 0.0001**
Score 2				204 (88)	27 (12)	
Score 3				42 (64)	24 (36)	
**Oestrogen receptor**						
Negative	287 (62)	180 (38)	59.4 ** < 0.0001**	127 (73)	47 (27)	49.2 ** < 0.0001**
Positive	1188 (79)	313 (21)		549 (92)	47 (8)	
**Progesterone receptor**						
Negative	63 (69)	293 (31)	39.1 ** < 0.0001**	199 (78)	57 (22)	39.5 ** < 0.0001**
Positive	838 (81)	200 (19)		471 (93)	37(7)	
**Human epidermal growth factor receptor 2 (HER2) status**						
Negative	1329 (77)	399 (23)	29.1 ** < 0.0001**	487 (90)	54 (10)	8.2 **0.017**
Positive	146 (61)	94 (39)		102 (81)	24 (19)	
**Triple-negative status**						
Non-triple negative	1270 (77)	381 (23)	21.3 <0.0001	***N/A***	***N/A***	***N/A***
Triple negative	205 (65)	112(35)				
**TP53 mutations**						
Wild type	531 (74)	188 (26)	6.9 **0.009**	***N/A***	***N/A***	***N/A***
Mutation	58 (61)	37 (39)				

*P* values in bold means statistically significant, N/A not available

TCGA BC dataset showed similar significant results in addition to the association with nucleolar score 3 (*p* < 0.0001) [4] (Table 1). The association between *NOP10* mRNA and aggressive features of the tumour were also demonstrated and confirmed in the Breast Cancer Gene Expression Miner v4.3 database (Supplementary Fig. S1a-h).

### NOP10 protein expression

NOP10 protein expression was observed mainly in the nucleus and nucleolus of invasive tumour cells, with expression levels varying from negative to strong (Fig. 1b, c and d). Excellent concordance was observed between the two observers (ICC = 0.803, *p* < 0.0001 for nuclear expression & ICC = 0.721, *p* < 0.0001 for nucleolar expression). High NOP10 nuclear and nucleolar expression (cut-off > 120 H-score & > 0 H-score, respectively) were displayed in 847/1081 (78%) and 58/1081 (5%) of BC cases.

High expression of NOP10 protein whether in the nucleus or in the nucleoli showed a significant association with aggressive characteristics including; higher tumour grade (*p* < 0.0001), poor NPI (*p* = 0.006 & *p* = 0.002), higher mitotic scores (*p* = 0.0003 & *p* = 0.0002), higher nuclear pleomorphism (*p* < 0.0001 & *p* = 0.017) and higher nucleolar score (*p* = 0.003 & *p* < 0.0001). High NOP10 nucleolar protein expression was significantly associated with receptor negativity (ER-, PR- and TNBC phenotype) (all *p* < 0.0001) (Table 2).

**Table 2:** Clinicopathological associations of NOP10 nuclear & nucleolar protein expression in Nottingham cohort

Parameter	NOP10 nuclear expression		Chi- square (χ2) (*p*-value)	NOP10 nucleolar expression		Chi- square (χ2) (*p*-value)
	Low N (%)	High N (%)		Low N (%)	High N (%)	
**Patient age**						
≤ 50 years	83 (21)	317 (79)	0.32 0.58	342 (94)	22 (6)	0.49 0.48
> 50 years	151 (22)	529 (78)		681 (95)	36 (5)	
**Tumour size**						
≤ 2 cm	143 (21)	523 (79)	0.02 0.9	609 (95)	29 (5)	2.11 0.14
> 2 cm	90 (22)	323 (78)		412 (93)	29 (7)	
**Axillary nodal stage**						
Stage 1	138 (21)	521 (79)	0.82 0.66	619 (94)	40 (6)	1.60 0.44
Stage 2	72 (23)	236 (77)		295 (96)	13 (4)	
Stage 3	23 (21)	89 (79)		107 (95)	5 (5)	
**Lympho-vascular invasion**						
Negative	157 (21)	598 (79)	0.95 0.33	711 (94)	44 (6)	1.10 0.31
Positive	76 (24)	248 (76)		310 (96)	14 (4)	
**Histological subtypes**						
Lobular	26 (35)	48 (65)	22.10 **0.0002**	74 (100)	0 (0)	19.80 **0.001**
Tubular	35 (32)	76 (68)		53 (96)	2 (4)	
Non-specific type(NST)	140 (18)	633 (82)		134 (100)	0 (0)	
Mixed NST and lobular	15 (28)	39 (72)		760 (96)	32 (4)	
Other special type*	18 (26)	51 (74)		62 (89)	8 (11)	
**Nottingham prognostic index (NPI)**						
Good	42 (22)	152 (78)	10.40 **0.006**	188 (96)	8 (4)	12.40 **0.002**
Moderate	111 (28)	486 (72)		573 (95)	32 (5)	
Poor	79 (19)	201 (81)		312 (99)	2 (1)	
**Tumour grade**						
Grade 1	36 (53)	32 (47)	44.90 ** < 0.0001**	68 (100)	0 (0)	19.40 ** < 0.0001**
Grade 2	95 (22)	339 (78)		423 (97)	11 (3)	
Grade 3	102 (18)	476 (82)		531 (92)	47 (8)	
**Nuclear pleomorphism**						
Score 1	4 (67)	2 (33)	18.7 ** < 0.0001**	6 (100)	0 (0)	8.2 **0.017**
Score 2	67 (29)	162 (71)		225 (98)	4 (2)	
Score 3	158 (19)	676 (81)		780 (93)	54 (7)	
**Mitosis**						
Score 1	114 (27)	306 (73)	16.7 **0.0003**	412 (98)	8 (2)	17.4 **0.0002**
Score 2	51 (22)	184 (78)		219 (93)	16 (7)	
Score 3	64 (15)	348 (85)		378 (92)	34 (8)	
**Tubule formation**						
Score 1	16 (47)	18 (53)	13.9 **0.001**	34 (100)	0 (0)	6.1 **0.047**
Score 2	60 (22)	217 (78)		268 (97)	9 (3)	
Score 3	153 (20)	605 (80)		709 (93)	49 (7)	
**Nucleolar score**						
Score 1	88 (27)	234 (73)	11.9 **0.003**	341 (99)	2 (1)	98.2 ** < 0.0001**
Score 2	78 (19)	331 (81)		416 (99)	3 (1)	
Score 3	32 (16)	171 (84)		173 (84)	32 (16)	
**Oestrogen receptor**						
Negative	45 (19)	193 (81)	1.4 0.25	207 (87)	31 (13)	35.3 ** < 0.0001**
Positive	189 (22)	654 (78)		816 (97)	27 (3)	
**Progesterone receptor**						
Negative	101 (21)	374(79)	0.04 0.84	433 (91)	42 (9)	21.4 ** < 0.0001**
Positive	131 (22)	471 (78)		587 (97)	15 (3)	
**Human epidermal growth factor receptor 2 (HER2) status**						
Negative	202 (22)	719 (78)	0.3 0.58	874 (95)	47 (5)	0.8 0.4
Positive	32 (20)	128 (80)		149 (93)	11 (7)	
**Triple negative status**						
Non-triple negative	199 (22)	702 (78)	0.6 0.432	867 (96)	34 (4)	27.1 ** < 0.0001**
Triple negative	35 (19)	145 (81)		156 (87)	24 (13)	

^*^Other special types include tubular, mucinous, cribriform, papillary, micropapillary

*P* values in bold means statistically significant

### NOP10 and other related biomarkers

The correlation of *NOP10* mRNA with other genes in H/ACA snoRNPs was investigated using the METABRIC and TCGA datasets. *NOP10* was positively associated with *DKC1* (correlation coefficient (r) = 0.082 & 0.151, *p* = 0.0002 & *p* < 0.0001), *GAR1* (r = 0.264 & 0.413, both *p* < 0.0001), and *NHP2* (r = 0.214 & 0.447, both *p* < 0.0001) in METABRIC and TCGA, respectively. Moreover, there was a significant association with the regulatory gene *MYC* (r = 0.313 & 0.092, *p* < 0.0001 & *p* = 0.007) (correlation matrix of *NOP10* with other related genes in snoRNPs is shown in Supplementary Fig. S2a & b). High *NOP10* mRNA expression was associated with tumours exhibiting *TP53* mutations (*p* = 0.009, Table 1) in the METABRIC cohort.

At the protein level, there was a significant positive correlation between NOP10 and DKC1 nuclear expression (r = 0.284, *p* < 0.0001).

### NOP10 and patients’ outcome

**Fig. 2:**
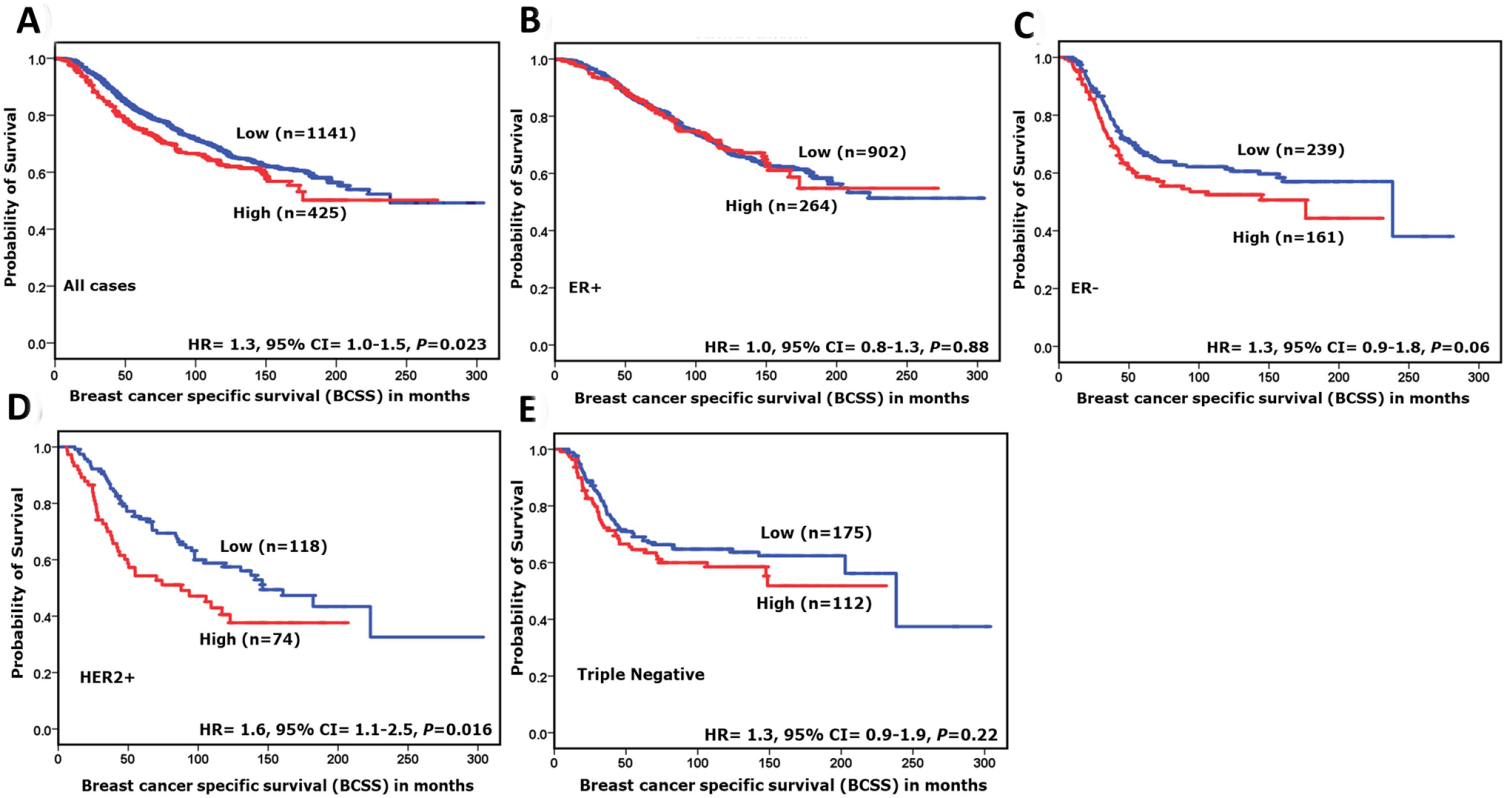
*NOP10* mRNA expression and breast-cancer-specific survival (BCSS) in **a** All cases, **b** oestrogen receptor (ER)-positive tumours **and c** ER-negative tumours. **d** Human epidermal growth factor receptor 2 (HER2 +) tumours. **e** Triple-negative tumours in the METABRIC cohort

In the METABRIC cohort, high *NOP10* mRNA expression was associated with poor outcome in terms of shorter BCSS (HR = 1.3, 95%CI = 1.0–1.5; *p* = 0.023). When the cohort was stratified according to the molecular subtypes, high *NOP10* mRNA expression was predictive of shorter BCSS in HER2 enriched tumours (HR = 1.6, 95%CI = 1.1–2.5; *p* = 0.016) but not in other classes (Fig. 2a–e). Similarly, in the TCGA cohort, there was an association between high *NOP10* mRNA expression and poor patient outcome in all cases and in the HER2 + tumours (HR = 1.8, 95%CI = 1.1–3.1; *p* = 0.035 & HR = 3.7, 95%CI = 1.2–11.5; *p* = 0.022), (Supplementary Fig. S3a–d).

**Fig. 3:**
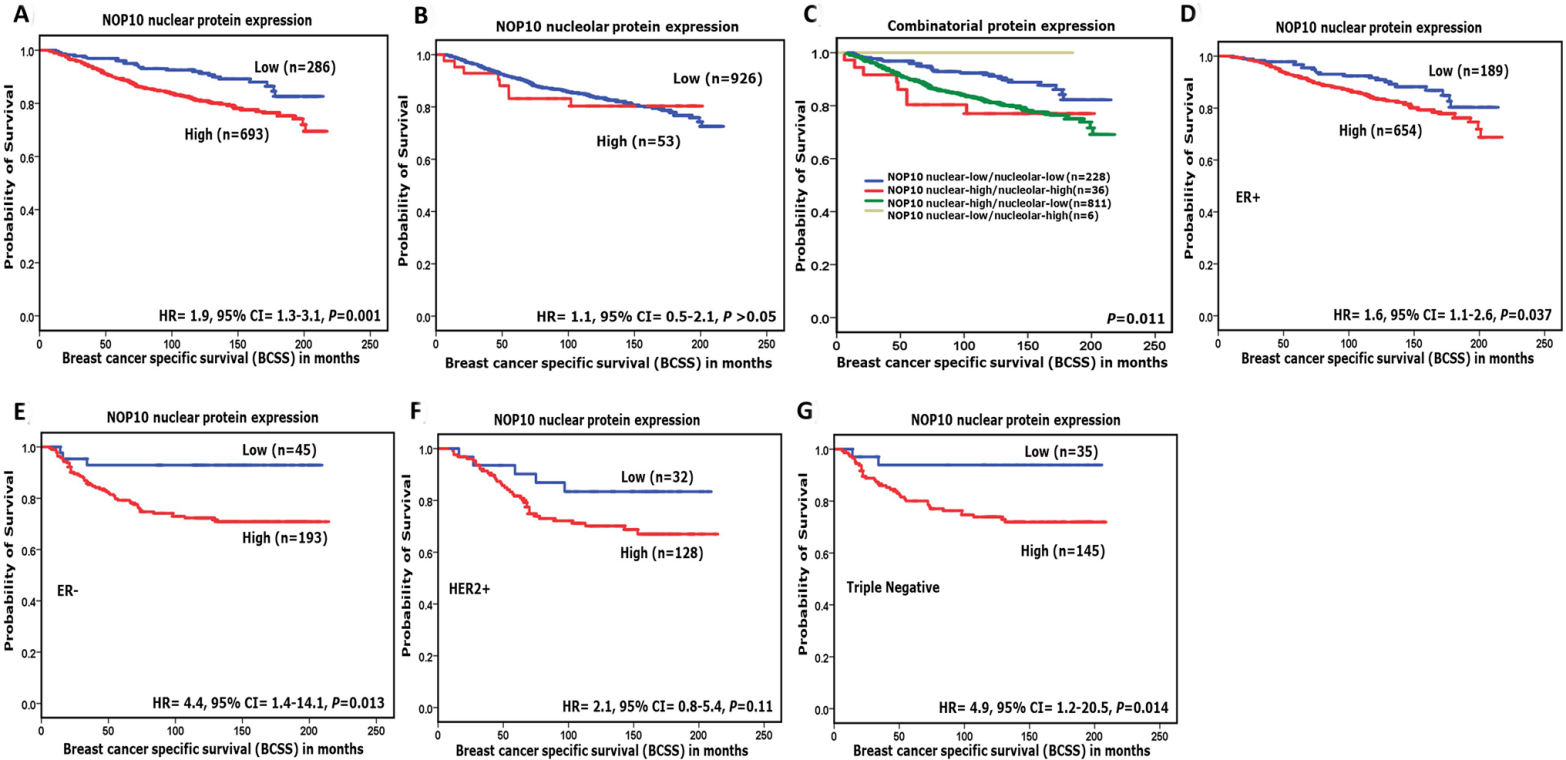
NOP10 protein expression and breast-cancer-specific survival (BCSS). **a** NOP10 nuclear expression and BCSS. **b** NOP10 nucleolar expression and BCSS. **c** combinatorial NOP10 protein expression and BCSS. **d** NOP10 and BCSS in oestrogen receptor (ER)-positive tumours. **e** NOP10 and BCSS in (ER)-negative tumours. **f** NOP10 and BCSS human epidermal growth factor receptor 2-positive (HER2 +) tumours. **g** NOP10 and BCSS of triple-negative tumours in Nottingham cohort

At the protein level, high NOP10 nuclear protein expression was significantly associated with shorter BCSS (HR = 1.9, 95%CI = 1.3–3.1; *p* = 0.001) and shorter DMFS (HR = 1.8, 95%CI = 1.3–2.7; *p* = 0.001) in the whole cohort, and in the TNBC class (HR = 4.9, 95%CI = 1.2–20.5; *p* = 0.014 and HR = 5.3, 95%CI = 1.3–21.8; *p* = 0.01) for BCSS and DMFS, respectively) (Fig. 3a–g and Supplementary Fig. S4a-e).

Combinatorial NOP10 protein expression groups [low nuclear/low nucleolar, high nuclear/low nucleolar, low nuclear/high nucleolar and high nuclear/high nucleolar expression] showed a significant difference in BCSS among these groups with the high nuclear/high nucleolar NOP10 expression group showing the worst outcome (*p* = 0.011) (Fig. 3c).

In multivariate Cox regression models including other prognostic covariates (tumour size, grade and nodal stage), *NOP10* mRNA was an independent predictor of shorter BCSS (*p* = 0.04, HR 1.3, 95% CI = 1.0–1.7) (Supplementary Table S3). High NOP10 nuclear and combinatorial protein expression (high nuclear/high nucleolar) were independent predictors of poor prognosis in all cases (*p* = 0.002, HR 1.9, 95% CI = 1.3 –2.9 & *p* = 0.005, HR 1.3, 95% CI = 1.1 – 1.6) and in TNBC cases only (*p* = 0.014, HR 5.9, 95% CI = 1.4–24.9 & *p* = 0.04, HR 1.6, 95% CI = 1.1–2.6) as shown in Table 3.

**Table 3:** Multivariate Cox regression analysis results for predictors of Breast-Cancer-Specific Survival in Nottingham cohort

Models	Parameters	All cases				Triple-negative tumours			
		Hazard ratio (HR)	95% confident interval (CI)		Significance *p*-value	Hazard ratio (HR)	95% confident interval (CI)		Significance *p*-value
			Lower	Upper			Lower	Upper	
(A)	NOP10 nuclear expression	1.9	1.3	2.9	**0.002**	5.9	1.4	24.9	**0.014**
	Tumour size	1.6	1.2	2.2	**0.002**	1.3	0.7	2.4	0.465
	Tumour stage	1.8	1.5	2.2	** < 0.0001**	1.9	1.3	3.2	**0.004**
	Tumour grade	1.9	1.4	2.6	**0.00016**	0.9	0.4	2.6	0.969
(B)	NOP10 nucleolar expression	0.9	0.9	1.0	0.29	0.4	0.05	2.7	0.329
	Tumour size	1.5	1.1	1.9	**0.004**	1.3	0.7	2.4	0.425
	Tumour stage	1.9	1.6	2.2	** < 0.0001**	1.9	1.2	2.8	**0.002**
	Tumour grade	2.0	1.6	2.6	** < 0.0001**	0.9	0.5	2.1	0.944
(C)	NOP10 combinatorial protein expression	1.3	1.1	1.6	**0.005**	1.6	1.1	2.6	**0.04**
	Tumour size	1.6	1.2	2.2	**0.002**	1.4	0.7	2.5	0.332
	Tumour stage	1.8	1.5	2.2	** < 0.0001**	1.8	1.2	2.7	**0.002**
	Tumour grade	1.9	1.4	2.6	** < 0.0001**	0.8	0.4	1.7	0.581

*P* values in bold means statistically significant

**Fig. 4:**
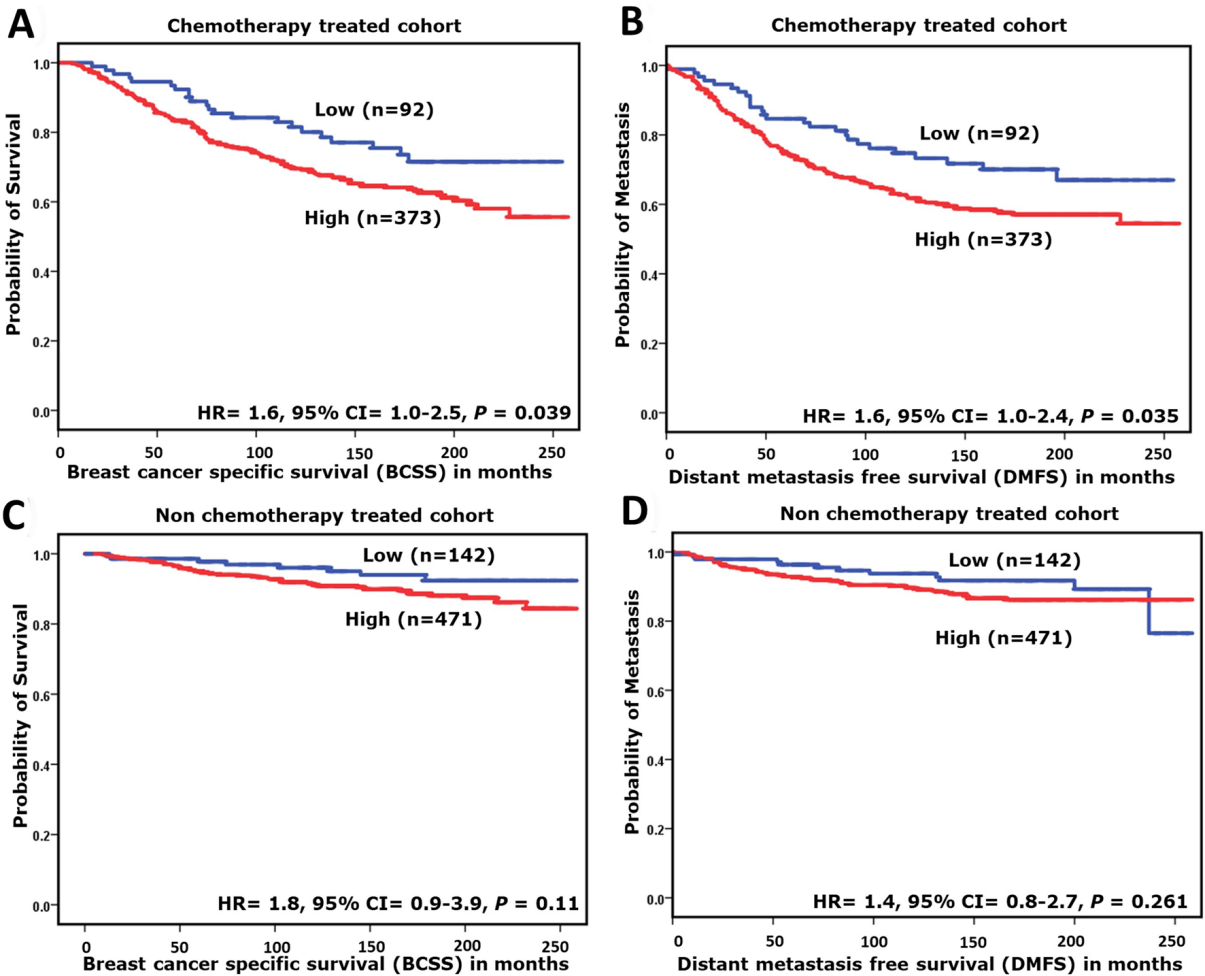
Kaplan–Meier survival plots showing the association between the expression of NOP10 nuclear protein expression and breast cancer-specific survival (BCSS) and Distant metastasis-free survival (DMFS), in chemotherapy-treated **a** and **b** and non-treated **c** and **d** cohorts

When patients were classified based on therapy, high NOP10 protein expression in patients who received chemotherapy was significantly associated with shorter BCSS (HR = 1.6, 95%CI = 1.0–2.5; *p* = 0.039) and higher risk of early distant metastasis (HR = 1.6, 95%CI = 1.0–2.4; *p* = 0.035). However, such association was not observed in those who did not receive systemic chemotherapy (Fig. 4a-d).

In the chemotherapy-treated cohort, high NOP10 nuclear expression was predictive of a higher risk of death from breast cancer (*p* = 0.028, HR 1.6, 95% CI = 1.1–2.6) and development of distant metastasis (*p* = 0.02, HR 1.6, 95% CI = 1.1–2.5), independent of tumour size, nodal stage and tumour grade (Table 4).

**Table 4:** Multivariate Cox regression analysis results for predictors of (A) Breast-Cancer-Specific Survival (BCSS) & (B) Distant metastasis-free survival (DMFS) in chemotherapy-treated cohort

Parameters	Chemotherapy-treated cohort							
	Breast-cancer-specific survival (BCSS)				Distant metastasis-free survival (DMFS)			
	Hazard ratio (HR)	95% confident interval (CI)		Significance *p*-value	Hazard ratio (HR)	95% confident interval (CI)		Significance *p*-value
		Lower	Upper			Lower	Upper	
NOP10 nuclear expression	1.6	1.1	2.6	**0.028**	1.6	1.1	2.5	**0.02**
Tumour size	1.1	0.9	1.2	0.32	1.1	0.9	1.2	0.45
Tumour stage	1.3	1.1	1.6	**0.009**	1.4	1.2	1.7	**0.001**
Tumour grade	0.9	0.6	1.3	0.56	0.8	0.6	1.1	0.11

*P* values in bold means statistically significant

## Discussion

Ribosome production is an essential process during neoplastic transformation. Regulation of cellular growth and proliferation mainly depends on the rate of ribosomal biogenesis. Cancer cells required increased production of ribosomes to sustain their high demand for protein synthesis. Protein synthesis requires ribosomal RNAs besides the protein components of the translation machinery [6].

The underlying molecular mechanisms of ribosomal modifications in various human diseases including malignancy are diverse and not fully understood [32–34]. Previous studies on neuroblastoma and lymphocytic leukemia demonstrated that snoRNP signature displayed highly significant prognostic value and was an independent predictor of poor prognosis through its effect on genomic stability and telomere maintenance [10, 11].

NOP10 is one of four essential protein components of H/ACA snoRNPs, which plays a potential role in facilitating the modification and stabilization of ribosomal RNAs. In addition, NOP10 is required for optimal enzymatic activity [35].

To further explore the potential value of snoRNPs family, we investigated for the first time, to the best our knowledge, the prognostic and predictive significance of NOP10 in BC using genomic, transcriptomic, and proteomic data of large BC cohorts. NOP10 protein expression was assessed in the nuclei and nucleoli of BC cells to determine its association with the nucleolar score [4].

The number and size of nucleoli as a consequence of its elevated activity in ribosomal biogenesis has been widely used as a prognostic marker of aggressive cancer [4, 36]. In the current study, a significant association between high NOP10 at both level protein (whether in nucleus or nucleoli) and mRNA expression (transcriptomic) with the nucleolar score (phenotype) was observed. These findings may support the speculation that NOP10 expression correlates with nucleoli appearance and size through its functional role in ribosomal RNA modification.

We revealed the significant association between the high expression of NOP10, at both protein and mRNA levels, and poor prognostic clinicopathological parameters and worse survival supporting its importance in BC progression. These data are in accordance with a study which showed alterations in *NOP10* mRNA are associated with poor prognosis in endometrial cancer [37]. Moreover, we demonstrated a significant association between *NOP10* mRNA expression and CN variation which supports the idea that hypotheses the diverging expression of snoRNPs could be closely associated with an overall elevation in genetic aberrations in BC [10, 38]. We reported a higher percentage in NOP10 expression at the proteomic level compared to the mRNA level, this could be related to sample fixation, age of stored samples or the specificity of antigen retrieval technique [39, 40]. Also, transcriptomic data reflects the mRNA level of all cells within the tissue samples either tumour cells or other surrounding cells.

We also evaluated the association of NOP10 and BC subtypes. NOP10 protein expression was significantly associated with shorter DMFS and BCSS in TNBC, while mRNA predicted poor outcome in HER2+ tumours. Such discrepancies might be attributed to the differences in the number of cases in each subtype between the Nottingham and METABRIC cohorts or might be due to tumour-specific differences in NOP10 mRNA/protein stability or post-transcriptional regulation of NOP10 expression.

NOP10 protein expression was independent prognostic markers in all cases and in TNBC, which may have potential clinical relevance in improving survival rate prediction. TNBC is highly malignant, prone to metastasis and relapse, and significantly correlated with a poorer prognosis and a greater risk of mortality comparing to other BC subtypes [41, 42]. NOP10 elevation is doubtlessly attributable to TNBC aggressive character through its heavier protein requirements for cell survival and proliferation which is compatible with increased rRNA synthesis [43]. This implies that NOP10 plays a role in tumourigenic pathways and could be a marker of poor prognosis in TNBC.

Since high expression of NOP10 was associated with worse prognostic features and outcome in patients with TNBC subtype, we hypothesized that NOP10 may play an important role in response to chemotherapy. Our findings showed that patients with high NOP10 expression showed poorer outcome than those with low levels of NOP10 even when chemotherapy was received which indicates that NOP10 could potentially contribute to chemotherapy resistance. These findings suggest that assessment of NOP10 expression prior to adjuvant treatment could predict the chemotherapy resistance and eventually tumour relapse.

*NOP10* plays a critical role for the stability of the domain as well as in the assembly and integrity of H/ACA snoRNPs complexes (*DKC1, NHP2, NOP10 & GAR1*), where they implicated mainly in the isomerization of uridine to pseudouridine thereby, promote the folding and stabilization of RNAs, such as the local RNA structure in the ribosome [44]. The current study confirmed the correlation among the gene expressions of all H/ACA ribonucleoproteins. High NOP10 protein expression was correlated with the high level of DKC1, indicating a system of functional coupling between these biomarkers at the protein level in isomerization and stabilization of RNAs.

Biogenesis of H/ACA snoRNPs is regulated in response to high demands for protein synthesis and c-*Myc* plays a crucial role in regulating cellular growth, size, and protein synthesis. Our study revealed a positive significant relationship between *NOP10* and c-*Myc.* Therefore, it can be speculated that modulation of *c-Myc* transcriptional activity may regulate *NOP10* expression in order to fulfil increased demands for protein synthesis that is highly required to maintain the proliferation and self-renewal of tumour cells [45–47].

*TP53* mutations were also highly prevalent in breast tumours where there was high *NOP10* mRNA expression. Marcel et al*.* have demonstrated that the level of rRNA methylation usually increased in cancer cells with dysfunctional p53, substantially elucidating that rRNA modifications contribute to the tumourigenic process. The high level of rRNA methylation resulted in initiating protein translation through a process called internal ribosome entry sequences (IRESs) which resulted in products that promote tumour development (The insulin-like growth factor 1 (*IGF-1R*), *c-Myc*, Vascular endothelial growth factor A (*VEGF-A*) and acidic fibroblast growth factor (*FGF1*)) [47–49].

Despite the remarkable results this study presents, there are some identified limitations. The subjectivity of the semi-quantitative H-score method, that has been used to score the sections, is one of our study weaknesses. It was aimed to reduce the impact of this limitation by allowing two well-trained observers to score about 10% of the cores to ensure the reproducibility and liability of the procedure. On the other hand, using TMA could underestimate the role of tumour heterogeneity.

In conclusion, our study revealed the prognostic and predictive importance of NOP10 in BC. NOP10 was associated with poor prognostic characteristics and poor survival outcome. Overexpression of NOP10 appears to play a role in the progression of TNBC and is potentially predictive for selecting patients who might develop resistance to chemotherapy. Thus, it could act as a potential prognostic marker and a therapeutic target. Functional assessment is warranted to reveal the specific role played by NOP10 in the BC, especially in the highly proliferative molecular subtypes.

## Electronic supplementary material

Below is the link to the electronic supplementary material.

Electronic supplementary material 1 (DOCX 1266 kb)

## Data Availability

The authors confirm the data that have been used in this work are available on reasonable request.

## References

[1] Siegel RL, Miller KD (2018). Cancer statistics. A Cancer Journal for Clinicians.

[2] Rakha EA, El-Sayed ME, Lee AH, Elston CW, Grainge MJ, Hodi Z, Blamey RW, Ellis IO (2008). Prognostic significance of Nottingham histologic grade in invasive breast carcinoma. Journal of Clinical Oncology : Official Journal of the American Society of Clinical Oncology.

[3] Derenzini M, Trerè D, Pession A, Govoni M, Sirri V, Chieco P (2000). Nucleolar size indicates the rapidity of cell proliferation in cancer tissues. J Pathol.

[4] Elsharawy KA, Toss MS, Abuelmaaty SR, Ball G, Green AR, Aleskandarany MA, Dalton LW, Rakha EA (2019). Prognostic Significance of Nucleolar Assessment in Invasive Breast Cancer. Histopathology.

[5] Derenzini M, Montanaro L, Treré D (2009). What the nucleolus says to a tumour pathologist. Histopathology.

[6] Montanaro L, Trere D, Derenzini M (2008). Nucleolus, ribosomes, and cancer. The American journal of pathology.

[7] Kiss T (2001). Small nucleolar RNA-guided post-transcriptional modification of cellular RNAs. The EMBO journal.

[8] McMahon M, Contreras A, Ruggero D (2015). Small RNAs with big implications: new insights into H/ACA snoRNA function and their role in human disease. WIREs RNA.

[9] Murray JL, Sheng J, Rubin DH (2014). A role for H/ACA and C/D small nucleolar RNAs in viral replication. Mol Biotechnol.

[10] von Stedingk K, Koster J, Piqueras M, Noguera R, Navarro S, Pahlman S, Versteeg R, Ora I, Gisselsson D, Lindgren D, Axelson H (2013). snoRNPs Regulate Telomerase Activity in Neuroblastoma and Are Associated with Poor Prognosis. Translational oncology.

[11] Dos Santos PC, Panero J, Stanganelli C, Palau Nagore V, Stella F, Bezares R, Slavutsky I (2017). Dysregulation of H/ACA ribonucleoprotein components in chronic lymphocytic leukemia. PLoS ONE.

[12] Reichow SL, Hamma T, Ferré-D'Amaré AR, Varani G (2007). The structure and function of small nucleolar ribonucleoproteins. Nucleic Acids Res.

[13] Bachellerie JP, Cavaille J, Huttenhofer A (2002). The expanding snoRNA world. Biochimie.

[14] Mannoor K, Liao J (1826). Jiang F (2012) Small nucleolar RNAs in cancer. Biochem Biophys Acta.

[15] Liu B, Zhang J, Huang C, Liu H (2012). Dyskerin Overexpression in Human Hepatocellular Carcinoma Is Associated with Advanced Clinical Stage and Poor Patient Prognosis. PLoS ONE.

[16] Sieron P, Hader C, Hatina J, Engers R, Wlazlinski A, Müller M, Schulz WA (2009). DKC1 overexpression associated with prostate cancer progression. Br J Cancer.

[17] Schaner ME, Ross DT, Ciaravino G, Sørlie T, Troyanskaya O, Diehn M, Wang YC, Duran GE, Sikic TL, Caldeira S, Skomedal H, Tu I-P, Hernandez-Boussard T, Johnson SW, O'Dwyer PJ, Fero MJ, Kristensen GB, Børresen-Dale A-L, Hastie T, Tibshirani R, Mvd R, Teng NN, Longacre TA, Botstein D, Brown PO, Sikic BI (2003). Gene Expression Patterns in Ovarian Carcinomas. Mol Biol Cell.

[18] Grozdanov PN, Roy S, Kittur N, Meier UT (2009). SHQ1 is required prior to NAF1 for assembly of H/ACA small nucleolar and telomerase RNPs. RNA (New York, NY).

[19] Meier UT (2006). How a single protein complex accommodates many different H/ACA RNAs. Trends Biochem Sci.

[20] Darzacq X, Kittur N, Roy S, Shav-Tal Y, Singer RH, Meier UT (2006). Stepwise RNP assembly at the site of H/ACA RNA transcription in human cells. The Journal of cell biology.

[21] Curtis C, Shah SP, Chin SF, Turashvili G, Rueda OM, Dunning MJ, Speed D, Lynch AG, Samarajiwa S, Yuan Y, Graf S, Ha G, Haffari G, Bashashati A, Russell R, McKinney S, Langerod A, Green A, Provenzano E, Wishart G, Pinder S, Watson P, Markowetz F, Murphy L, Ellis I, Purushotham A, Borresen-Dale AL, Brenton JD, Tavare S, Caldas C, Aparicio S (2012). The genomic and transcriptomic architecture of 2,000 breast tumours reveals novel subgroups. Nature.

[22] Ciriello G, Gatza ML, Beck AH, Wilkerson MD, Rhie SK, Pastore A, Zhang H, McLellan M, Yau C, Kandoth C, Bowlby R, Shen H, Hayat S, Fieldhouse R, Lester SC, Tse GM, Factor RE, Collins LC, Allison KH, Chen YY, Jensen K, Johnson NB, Oesterreich S, Mills GB, Cherniack AD, Robertson G, Benz C, Sander C, Laird PW, Hoadley KA, King TA, Perou CM (2015). Comprehensive Molecular Portraits of Invasive Lobular Breast Cancer. Cell.

[23] El Ansari R, Craze ML, Miligy I, Diez-Rodriguez M, Nolan CC, Ellis IO, Rakha EA, Green AR (2018). The amino acid transporter SLC7A5 confers a poor prognosis in the highly proliferative breast cancer subtypes and is a key therapeutic target in luminal B tumours. Breast Cancer Res.

[24] Aleskandarany MA, Abduljabbar R, Ashankyty I, Elmouna A, Jerjees D, Ali S, Buluwela L, Diez-Rodriguez M, Caldas C, Green AR, Ellis IO, Rakha EA (2016). Prognostic significance of androgen receptor expression in invasive breast cancer: transcriptomic and protein expression analysis. Breast Cancer Res Treat.

[25] Rakha EA, Agarwal D, Green AR, Ashankyty I, Ellis IO, Ball G, Alaskandarany MA (2017). Prognostic stratification of oestrogen receptor-positive HER2-negative lymph node-negative class of breast cancer. Histopathology.

[26] Rakha EA, Elsheikh SE, Aleskandarany MA, Habashi HO, Green AR, Powe DG, El-Sayed ME, Benhasouna A, Brunet JS, Akslen LA, Evans AJ, Blamey R, Reis-Filho JS, Foulkes WD, Ellis IO (2009). Triple-negative breast cancer: distinguishing between basal and nonbasal subtypes. Clinical cancer research : an official journal of the American Association for Cancer Research.

[27] Abd El-Rehim DM, Ball G, Pinder SE, Rakha E, Paish C, Robertson JF, Macmillan D, Blamey RW, Ellis IO (2005). High-throughput protein expression analysis using tissue microarray technology of a large well-characterised series identifies biologically distinct classes of breast cancer confirming recent cDNA expression analyses. Int J Cancer.

[28] McCarty KS, McCarty KS (1984). Histochemical approaches to steroid receptor analyses. Semin Diagn Pathol.

[29] Camp RL, Dolled-Filhart M, Rimm DL (2004). X-Tile. A New Bio-Informatics Tool for Biomarker Assessment and Outcome-Based Cut-Point Optimization.

[30] McShane LM, Altman DG, Sauerbrei W, Taube SE, Gion M, Clark GM, Statistics Subcommittee of the NCIEWGoCD (2005). REporting recommendations for tumour MARKer prognostic studies (REMARK). Br J Cancer.

[31] Parker JS, Mullins M, Cheang MC, Leung S, Voduc D, Vickery T, Davies S, Fauron C, He X, Hu Z, Quackenbush JF, Stijleman IJ, Palazzo J, Marron JS, Nobel AB, Mardis E, Nielsen TO, Ellis MJ, Perou CM, Bernard PS (2009). Supervised risk predictor of breast cancer based on intrinsic subtypes. Journal of clinical oncology : official journal of the American Society of Clinical Oncology.

[32] Williams GT, Farzaneh F (2012). Are snoRNAs and snoRNA host genes new players in cancer?. Nat Rev Cancer.

[33] Valleron W, Ysebaert L, Berquet L, Fataccioli V, Quelen C, Martin A, Parrens M, Lamant L, de Leval L, Gisselbrecht C, Gaulard P, Brousset P (2012). Small nucleolar RNA expression profiling identifies potential prognostic markers in peripheral T-cell lymphoma. Blood.

[34] Valleron W, Laprevotte E, Gautier EF, Quelen C, Demur C, Delabesse E, Agirre X, Prosper F, Kiss T, Brousset P (2012). Specific small nucleolar RNA expression profiles in acute leukemia. Leukemia.

[35] Li S, Duan J, Li D, Yang B, Dong M, Ye K (2011). Reconstitution and structural analysis of the yeast box H/ACA RNA-guided pseudouridine synthase. Genes Dev.

[36] Donizy P, Biecek P, Halon A, Maciejczyk A, Matkowski R (2017). Nucleoli cytomorphology in cutaneous melanoma cells - a new prognostic approach to an old concept. Diagnostic pathology.

[37] Button L, Alnafakh R, Drury J, DeCruze S, Saretzki G, Adishesh M, Hapangama D (2019). P64 Examination of genes encoding telomerase associated proteins suggests a prognostic relevance for NHP2 and NOP10 in endometrial cancer. International Journal of Gynecologic Cancer.

[38] Lin P, Mobasher ME, Hakakian Y, Kakarla V, Naseem AF, Ziai H, Alawi F (2015). Differential requirements for H/ACA ribonucleoprotein components in cell proliferation and response to DNA damage. Histochem Cell Biol.

[39] Atkins D, Reiffen K-A, Tegtmeier CL, Winther H, Bonato MS, Störkel S (2004). Immunohistochemical Detection of EGFR in Paraffin-embedded Tumour Tissues: Variation in Staining Intensity Due to Choice of Fixative and Storage Time of Tissue Sections. J Histochem Cytochem.

[40] Saxby AJ, Nielsen A, Scarlett CJ, Clarkson A, Morey A, Gill A, Smith RC (2005). Assessment of HER-2 Status in Pancreatic Adenocarcinoma: Correlation of Immunohistochemistry, Quantitative Real-Time RT-PCR, and FISH With Aneuploidy and Survival. The American journal of surgical pathology.

[41] Cadoo KA, Fornier MN, Morris PG (2013) Biological subtypes of breast cancer current concepts and implications for recurrence patterns The quarterly journal of nuclear medicine and molecular imaging official publication of the Italian Association of Nuclear Medicine. AIMN) [and] the International Association of Radiopharmacology (IAR), [and] Section of the So 57 (4):312–321 24322788

[42] Gluz O, Liedtke C, Gottschalk N, Pusztai L, Nitz U, Harbeck N (2009). Triple-negative breast cancer–current status and future directions. Annals of oncology : official journal of the European Society for Medical Oncology.

[43] Putti TC, El-Rehim DMA, Rakha EA, Paish CE, Lee AHS, Pinder SE, Ellis IO (2005). Estrogen receptor-negative breast carcinomas: a review of morphology and immunophenotypical analysis. Mod Pathol.

[44] Ye K (2007). H/ACA guide RNAs, proteins and complexes. Curr Opin Struct Biol.

[45] van Riggelen J, Yetil A, Felsher DW (2010). MYC as a regulator of ribosome biogenesis and protein synthesis. Nat Rev Cancer.

[46] Wu CH, Sahoo D, Arvanitis C, Bradon N, Dill DL, Felsher DW (2008). Combined analysis of murine and human microarrays and ChIP analysis reveals genes associated with the ability of MYC to maintain tumourigenesis. PLoS Genet.

[47] Liang J, Wen J, Huang Z, Chen X-p, Zhang B-x, Chu L (2019). Small Nucleolar RNAs Insight Into Their Function in Cancer. Frontiers in Oncology.

[48] Marcel V, Ghayad SE, Belin S, Therizols G, Morel AP, Solano-Gonzalez E, Vendrell JA, Hacot S, Mertani HC, Albaret MA, Bourdon JC, Jordan L, Thompson A, Tafer Y, Cong R, Bouvet P, Saurin JC, Catez F, Prats AC, Puisieux A, Diaz JJ (2013). p53 acts as a safeguard of translational control by regulating fibrillarin and rRNA methylation in cancer. Cancer Cell.

[49] Marcel V, Catez F, Diaz J-J (2015). Ribosome heterogeneity in tumourigenesis: the rRNA point of view. Molecular & Cellular Oncology.

